# Supplementary material for: Establishment and range expansion of Dermacentor variabilis in the northern Maritimes of Canada: Community participatory science documents establishment of an invasive tick species
Source: PLoS One. 2023 Oct 13;18(10):e0292703. doi: 10.1371/journal.pone.0292703 (PMC10575507; doi:10.1371/journal.pone.0292703)
Supplement: S1 File — (PDF) [file pone.0292703.s001.pdf]

Supplemental Table 1. Tick recoveries from Nova Scotia from 2012-2021

		Tick life stage			Host		
year	Total[1]	female	male	immature	dog	human	other[2]
2012	1	1	0	0	0	1	
2013	15	14	1	0	0	15	0
2014	18	13	5	0	4	14	0
2015	65	42	23	0	31	32	2
2016	33	23	10	0	3	30	0
2017	67	35	32	0	16	51	0
2018	56	37	19	0	12	44	1
2019	87	48	37	2	19	52	6
2020	15	12	3	0	3	11	1
2021	20	15	5	0	0	20	0

1. Total ticks collected that year as donations to lab and through Geneticks commercial tick testing service.
2. Other = in house, on vegetation, cat

A

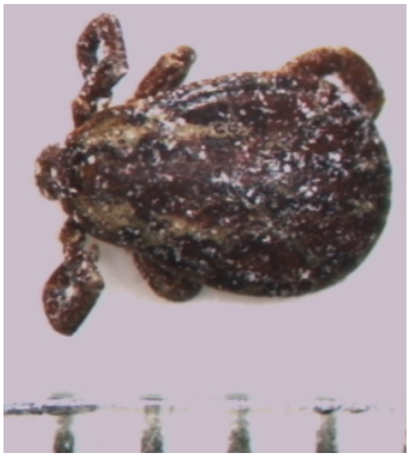

B

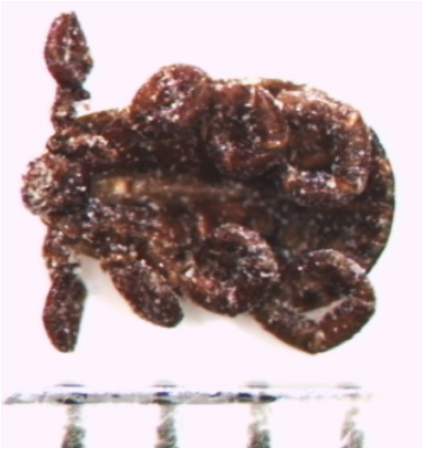

C

ATTTCTATAGAATTAATTATTTTTAGTTGGGGCAACT
AGAAAATATTATAAACTTTTTATATAATAAAATGACC
CATTATTAATGATTATTTGAGAAAAATACTCTAGGG
ATAACAGCGTAATAATTTTTGATAGAACCTATAGAC
AAAATAGTTTGCGACCTCGATGTTGGATTAGGATAC
TTTTTTAATGAAGATGTTAAAAAAAGAAGTTTGTTC
AACTTTTAAAGTCCTACTTGATCTGAGTTTCAGACC
GGAA

D

GNGACAACTTCTTTTTTTACATCTTCATTAAAAAAGTATCCTAATC
CAACATCGAGGTCGCAAACTATTTTGTCTATAGGTTCTATCAAAA
ATTATTACGCTGTTATCCCTAGAGTATTTTTCTCAAATAATCATTAA
TAATGGGTCATTTTATTATATAAAAAGTTTATAATATTTTCTAGTTG
CCCCAACTAAAAATAATTAATTTCTTATAAGAAATTAATTATTTTAA
AATTCATAGGGTCTCCTTGCCCAAAA

E

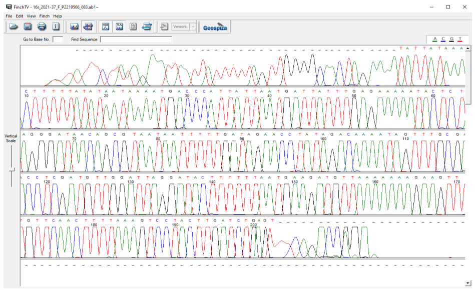

F

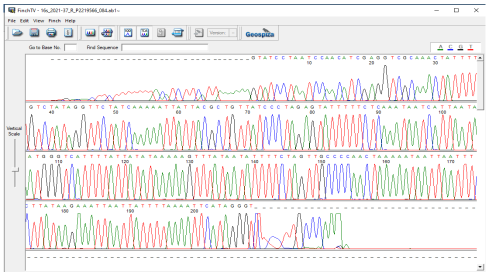

G

Description	Scientific Name	Max Score	Total Score	Query Cover	E value	Per. Ident	Acc. Len	Accession
Dermacentor variabilis isolate TamMex large subunit ribosomal RNA gene, partial sequence; mitochondrial	Dermacentor var...	375	375	100%	8e-100	100.00%	454	MK742796.1
Dermacentor variabilis isolate Dv0745_CO 16S ribosomal RNA gene, partial sequence; mitochondrial	Dermacentor var...	375	375	100%	8e-100	100.00%	454	MG834244.1
Dermacentor variabilis isolate Dv0743_CO 16S ribosomal RNA gene, partial sequence; mitochondrial	Dermacentor var...	375	375	100%	8e-100	100.00%	454	MG834242.1
Dermacentor variabilis isolate Dv0450_IN 16S ribosomal RNA gene, partial sequence; mitochondrial	Dermacentor var...	375	375	100%	8e-100	100.00%	454	MG834241.1
Dermacentor variabilis isolate Dv0449_IN 16S ribosomal RNA gene, partial sequence; mitochondrial	Dermacentor var...	375	375	100%	8e-100	100.00%	454	MG834240.1
Dermacentor variabilis isolate Dv0448_IN 16S ribosomal RNA gene, partial sequence; mitochondrial	Dermacentor var...	375	375	100%	8e-100	100.00%	454	MG834239.1

H

Description	Scientific Name	Max Score	Total Score	Query Cover	E value	Per. Ident	Acc. Len	Accession
Dermacentor variabilis isolate TJM355_ 16S ribosomal RNA gene, partial sequence; mitochondrial	Dermacentor var...	387	454	100%	4e-103	100.00%	490	KX673177.1
Dermacentor variabilis isolate TJM308-3-18_tick 16S ribosomal RNA gene, partial sequence; mitochondrial	Dermacentor var...	387	454	100%	4e-103	100.00%	244	KX673173.1
Dermacentor variabilis isolate KTTX5_tick 16S ribosomal RNA gene, partial sequence; mitochondrial	Dermacentor var...	387	534	100%	4e-103	100.00%	381	KX673168.1
Dermacentor variabilis mitochondrion mitochondrial 16S ribosomal RNA, mitochondrial gene, partial sequence	Dermacentor var...	387	454	100%	4e-103	100.00%	335	U14144.1
Dermacentor variabilis isolate ND_N128168B large subunit ribosomal RNA gene, partial sequence; mitochondrial	Dermacentor var...	381	449	100%	2e-101	99.52%	384	MK271157.1
Dermacentor variabilis isolate ND_N128168A large subunit ribosomal RNA gene, partial sequence; mitochondrial	Dermacentor var...	381	449	100%	2e-101	99.52%	384	MK271156.1

Supplemental Figure 1. Adult male *D variabilis*, 2021-37. A Dorsal view. B Ventral view. C *16S* sequence (Forward). D *16S* sequence (Reverse). E Chromatogram for the *16S* sequence (Forward) sequence. F Chromatogram for the *16S* sequence (Reverse) sequence. G Top blast results from NCBI genbank for the *16S* sequence (Forward) sequence. H Top blast results from NCBI genbank for the *16S* sequence (Reverse) sequence.

A

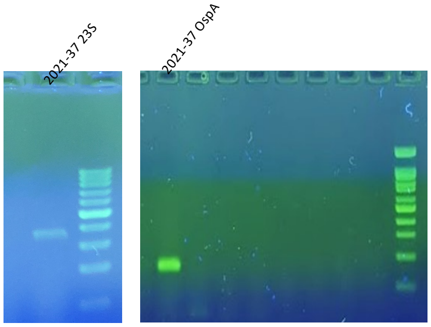

B

2021-37 23S:

GNNNAGCNTATTATAAGAAAACAAAAATTTATTCAATTTCTATAT
TAAGGTATATACGATTACTAACAATTACAACTTCATGCCATTGAG
TGTCGGGCAAATCCAAACTGAAATCTGCTTTATAGCATTTTGCT
TAGCTTTTTTGGGTTATTGTGGCAAACTTGTAACCTAAAACATA
AGAACGATAACGATTTAATGCAATCTTTGCCTTCCTCCAAATTAT
AAATGGCAGTCTTATCCGAATCCCTACCACTAAATACTGGCAAC
GATAACAAGGATTTAGGCTCGCTACAAATACTTGTCAA

2021-37 23S R:

CNTNGNGCGTATTAGTGGTAGGGATTCGGATAAGACTGCCATT
TATAATTTGGAGGAAGGCAAAGATTGCATTAAATCGTTATCGTT
CTTATGTTTTAGGTTACAAGGTTTGCCACAATAACCCAAAAAAG
CTAAGCAAAATGCTATAAAGCAGATTTCAGTTTGGATTTGCCCG
ACACTCAATGGCATGAAGTTGTAATTGTTAGTAATCGTATATACC
TTAATATAGAAATTGAATAAATTTTTGTTTTTCTTATTAATTATAG
CTTAAAACAGTATTGTCGTAATTAAAACAATGGGAATACATAAA

C

2021-37 OspA F:

GCTTNAGTTTTTCGCCGNTGTNTTTGAACGCCAACGGGC
CAGGGAAACACNTNGGATNCCNAATAGGGCCTGGTCNG
CCTGGTCGGGTGGGTTNATCNAGGTACNAAAACCCTTGA
CNCCTTCNACTCNAATGGNACCNAAA

2021-37 OspA R:

TTTGNCTNATTACCACCGACAGCCGACAGCCTATCGGTATC
GATGTGTTTCCNGGNCGTTNGCGTTNNAATANNANNGCG
GNAAAACANTNNTCCAAAGCAAGNTGGTTNCGGTCCGNN
NNAGGNNAA

D

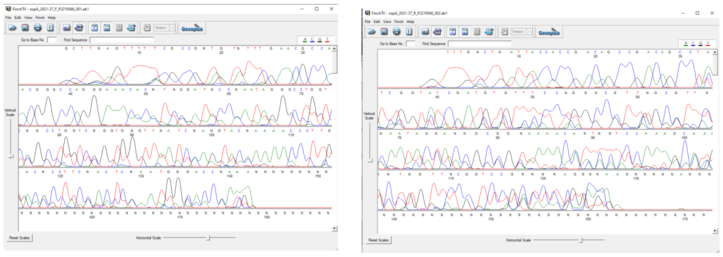

E

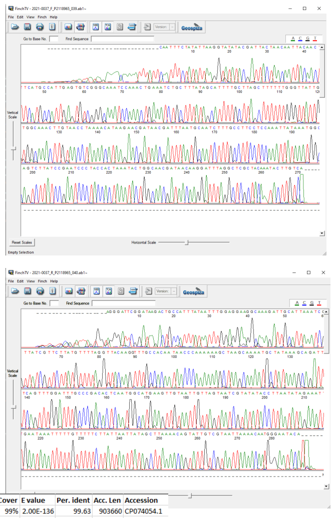

G

Description	Scientific Name	Max Score	Total Score	Query Cover	E value	Per. ident	Acc. Len	Accession
Borreliella burgdorferi strain B500 chromosome, complete genome	Borreliella burgdorferi	497	497	99%	2.00E-136	99.63	903660	CP074054.1
Borreliella burgdorferi strain B331 chromosome, complete genome	Borreliella burgdorferi	497	497	99%	2.00E-136	99.63	903654	CP017201.2
Borreliella burgdorferi isolate Bigbasin2_02 16S-23S ribosomal RNA intergenic spacer, partial sequence	Borreliella burgdorferi	497	497	99%	2.00E-136	99.63	857	MW862429.1
Borrelia burgdorferi strain BirdISlYU859 16S-23S ribosomal RNA intergenic spacer, partial sequence	Borreliella burgdorferi	497	497	99%	2.00E-136	99.63	812	GU142984.1
Borrelia burgdorferi strain B361 16S-23S ribosomal RNA intergenic spacer, partial sequence	Borreliella burgdorferi	497	497	99%	2.00E-136	99.63	791	EU377786.1
Borrelia burgdorferi strain 15912UT 16S-23S ribosomal RNA intergenic spacer, partial sequence	Borreliella burgdorferi	497	497	99%	2.00E-136	99.63	791	EU375816.1
Borrelia burgdorferi isolate HB19 16S-23S intergenic spacer, partial sequence	Borreliella burgdorferi	497	497	99%	2.00E-136	99.63	812	AY275205.1
Borrelia burgdorferi isolate 2-43a 16S-23S intergenic spacer, partial sequence	Borreliella burgdorferi	497	497	99%	2.00E-136	99.63	812	AY275203.1
Borrelia burgdorferi isolate B500 16S ribosomal RNA gene, and 16S-23S intergenic spacer, partial sequence; and tRNA-Ala gene, complete sequence	Borreliella burgdorferi	497	497	99%	2.00E-136	99.63	948	AF467866.1
Borrelia burgdorferi isolate 4-55 16S-23S intergenic spacer, partial sequence	Borreliella burgdorferi	492	492	99%	8.00E-135	99.27	812	AY275206.1
Borreliella burgdorferi isolate 110 16S-23S ribosomal RNA intergenic spacer, partial sequence	Borreliella burgdorferi	486	486	99%	4.00E-133	98.9	593	MN110103.1
Borreliella burgdorferi isolate 022 16S-23S ribosomal RNA intergenic spacer, partial sequence	Borreliella burgdorferi	486	486	99%	4.00E-133	98.9	907	MN110102.1

Description	Scientific Name	Max Score	Total Score	Query Cover	E value	Per. ident	Acc. Len	Accession
Borreliella burgdorferi strain B500 chromosome, complete genome	Borreliella burgdorferi	525	525	99%	8.00E-145	99.31	903660	CP074054.1
Borreliella burgdorferi strain B331 chromosome, complete genome	Borreliella burgdorferi	525	525	99%	8.00E-145	99.31	903654	CP017201.2
Borrelia burgdorferi isolate B500 16S ribosomal RNA gene, and 16S-23S intergenic spacer, partial sequence; and tRNA-Ala gene, complete sequence	Borreliella burgdorferi	525	525	99%	8.00E-145	99.31	948	AF467866.1
Borrelia burgdorferi strain BTW11 16S-23S ribosomal RNA intergenic spacer, partial sequence	Borreliella burgdorferi	514	514	99%	2.00E-141	98.63	945	JQ308238.1
Borrelia burgdorferi isolate B408 16S ribosomal RNA gene, and 16S-23S intergenic spacer, partial sequence; and tRNA-Ala gene, complete sequence	Borreliella burgdorferi	514	514	99%	2.00E-141	98.63	948	AF467864.1
Borrelia burgdorferi isolate B356 16S ribosomal RNA gene, and 16S-23S intergenic spacer, partial sequence; and tRNA-Ala gene, complete sequence	Borreliella burgdorferi	514	514	99%	2.00E-141	98.63	948	AF467861.1
Borrelia burgdorferi isolate B331 16S ribosomal RNA gene, and 16S-23S intergenic spacer, partial sequence; and tRNA-Ala gene, complete sequence	Borreliella burgdorferi	512	512	99%	6.00E-141	98.63	945	AF467862.1
Borrelia burgdorferi N40, complete genome	Borreliella burgdorferi N40	508	508	99%	8.00E-140	98.28	902191	CP002228.1
Borrelia burgdorferi isolate B348 16S ribosomal RNA gene, and 16S-23S intergenic spacer, partial sequence; and tRNA-Ala gene, complete sequence	Borreliella burgdorferi	508	508	99%	8.00E-140	98.28	948	AF467863.1
Borrelia burgdorferi strain N40 16S ribosomal RNA gene, partial sequence; and 16S-23S ribosomal RNA intergenic sequence, partial sequence	Borreliella burgdorferi N40	508	508	99%	8.00E-140	98.28	947	AF139516.1
Borrelia burgdorferi strain HWT37 16S-23S ribosomal RNA intergenic spacer, partial sequence	Borreliella burgdorferi	497	497	99%	2.00E-136	97.59	937	JQ356546.1
Borrelia burgdorferi strain HSP61 16S-23S ribosomal RNA intergenic spacer, partial sequence	Borreliella burgdorferi	497	497	99%	2.00E-136	97.59	959	JQ308249.1

Supplemental Figure 2. *Borrelia burgdorferi* sequence analysis of *D. variabilis* tick, 2021-37. A Images of agarose gels showing amplicons for *B. burgdorferi 23S* (left) and *OspA* (right) obtained from this tick. B Sequence of the *23S* amplicons, forward (top) and reverse (bottom). C Sequence of the *OspA* amplicons, forward (top) and reverse (bottom). D Chromatograms corresponding to the forward (left) and reverse (right) *23S* amplicons. E Chromatograms corresponding to the forward (top) and reverse (bottom) *OspA* amplicons. G Top blast results from NCBI genbank for the *23S* sequence (top) and *OspA* (bottom) sequences

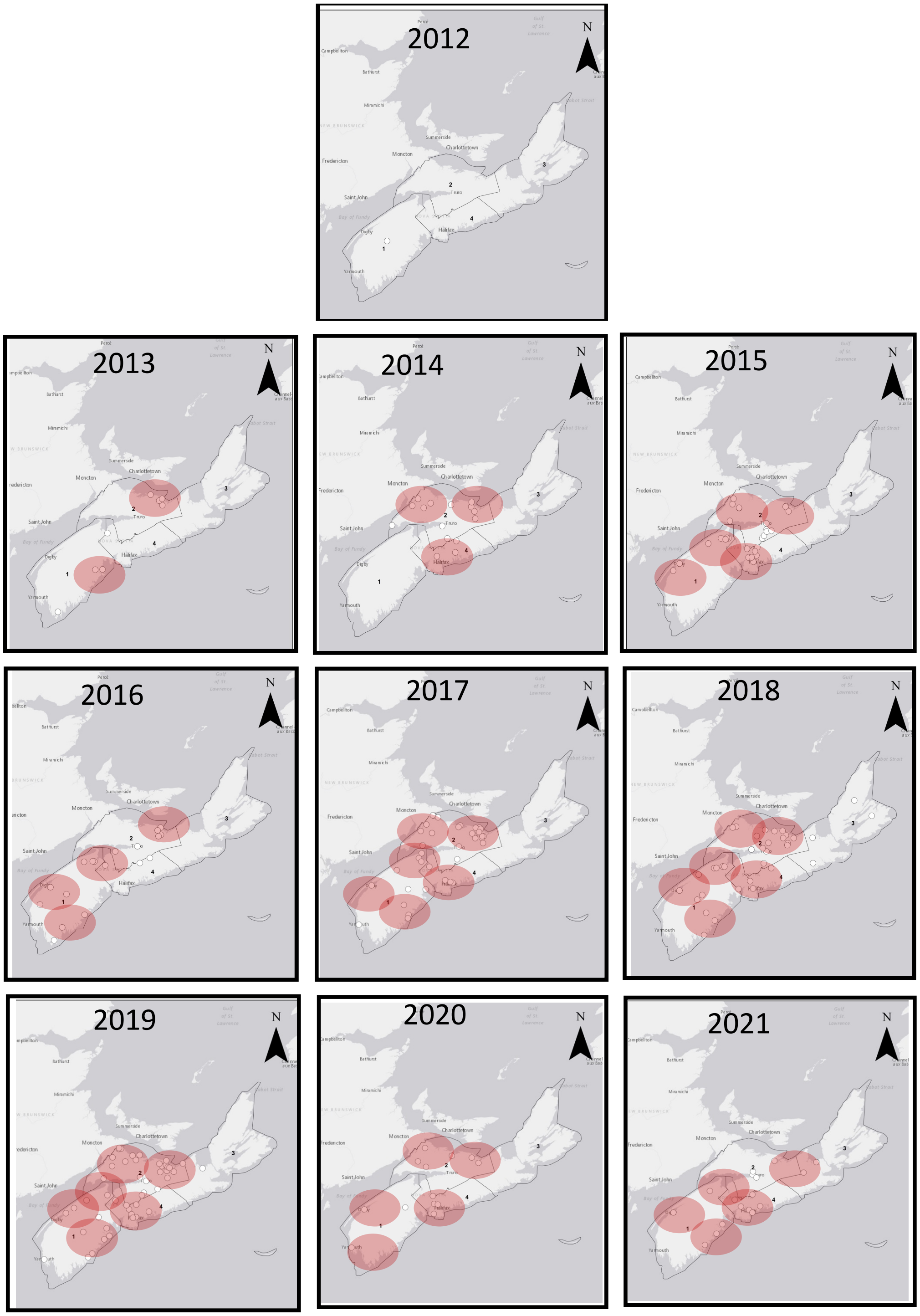
2012
2013
2014
2015
2016
2017
2018
2019
2020
2021
N
Campbellton
Bathurst
Miramichi
Summerside
Charlottetown
Moncton
Fredericton
Saint John
Bay of Fundy
Truro
Halifax
Digby
Yarmouth
1
2
3
4

Supplemental Figure 3 *D. variabilis* recoveries from Nova Scotia from 2012-2021. As of 2015, recoveries were extensive and constant with approximately 6 clusters (red dots) representing most of mainland Nova Scotia. Map templates were obtained from ArcGIS Online maps hosted by Esri.
