# Supplementary figures and images for: Establishment and range expansion of Dermacentor variabilis in the northern Maritimes of Canada: Community participatory science documents establishment of an invasive tick species
Source: PLoS One. 2023 Oct 13;18(10):e0292703. doi: 10.1371/journal.pone.0292703 (PMC10575507; doi:10.1371/journal.pone.0292703)

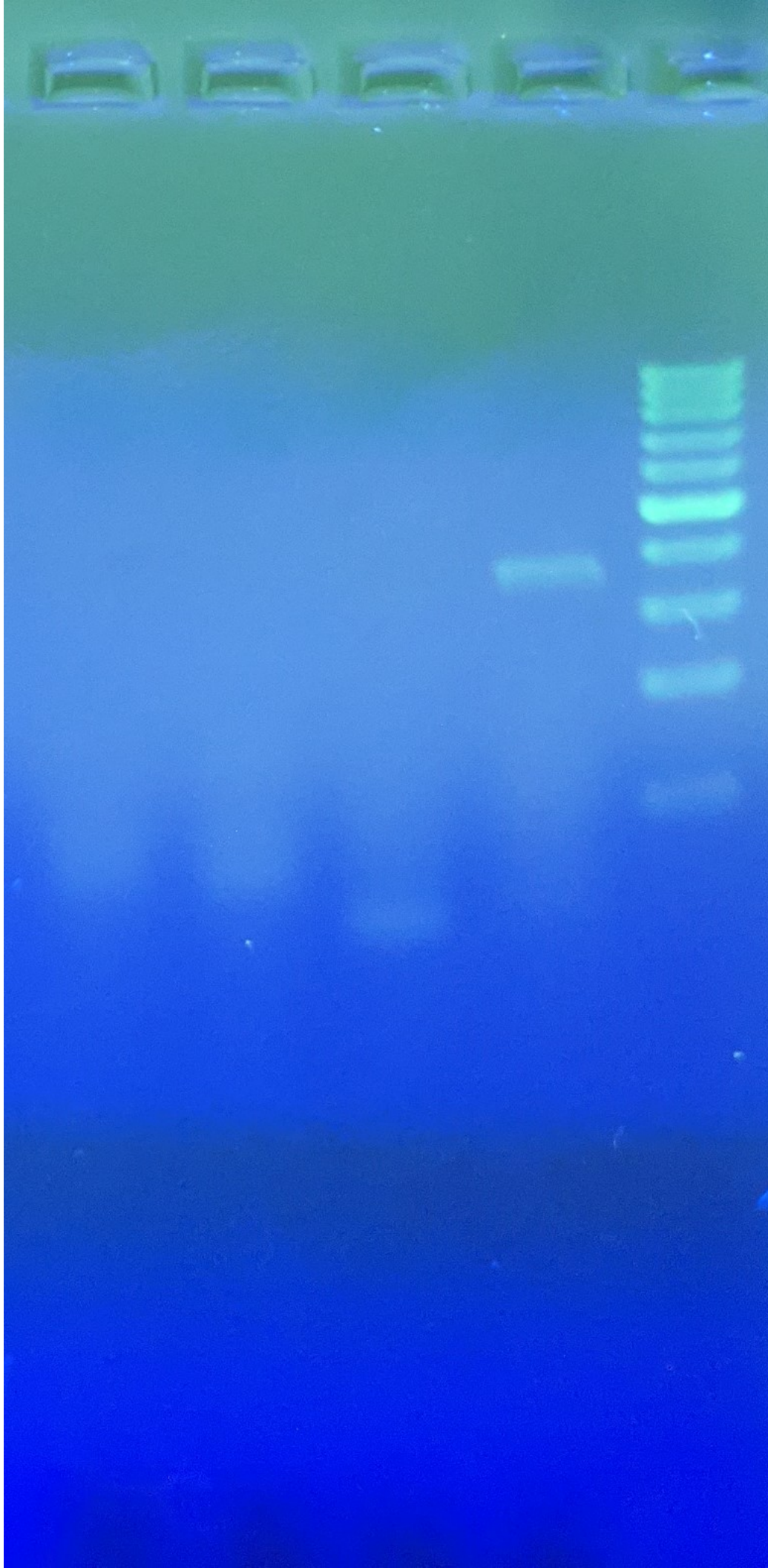

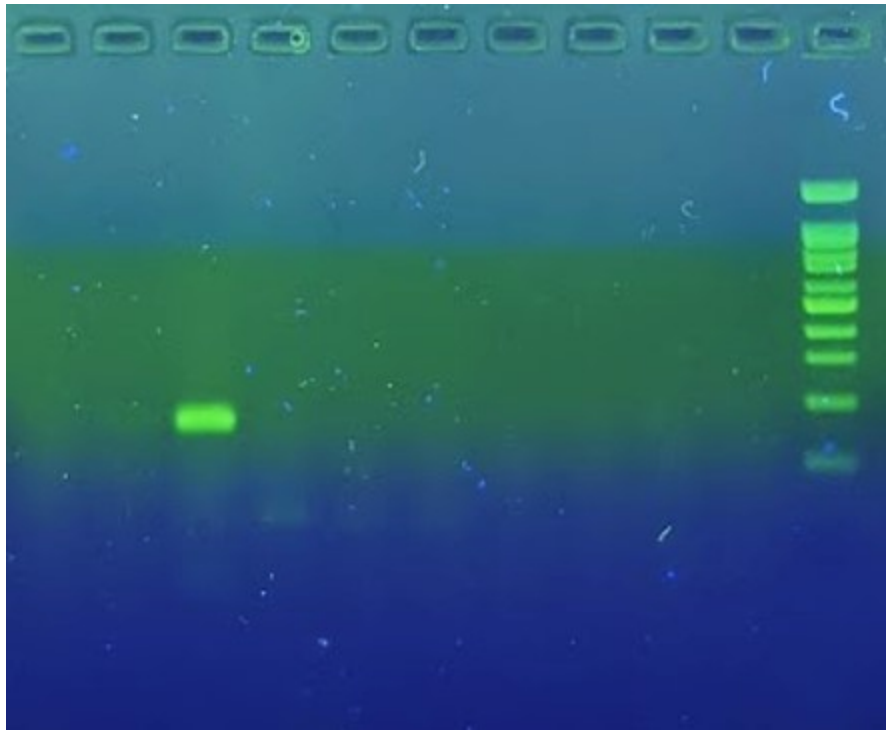

Supplement: S1 Raw images — (PDF) [file pone.0292703.s002.pdf]
